# X-linked lymphoproliferative syndrome in mainland China: review of clinical, genetic, and immunological characteristic

**DOI:** 10.1007/s00431-019-03512-7

**Published:** 2019-11-21

**Authors:** Tao Xu, Qin Zhao, Wenyan Li, Xuemei Chen, Xiuhong Xue, Zhi Chen, Xiao Du, Xiaoming Bai, Qian Zhao, Lina Zhou, Xuemei Tang, Xi Yang, Hirokazu Kanegane, Xiaodong Zhao

**Affiliations:** 1 Ministry of Education Key Laboratory of Child Development and Disorders, Children’s Hospital of Chongqing Medical University, Chongqing, China; 2 Chongqing Key Laboratory of Child Infection and Immunity, Children’s Hospital of Chongqing Medical University, Chongqing, China; 3 Division of Rheumatology and Immunology, Children’s Hospital of Chongqing Medical University, Chongqing, China; 4 Department of Child Health and Development, Graduate School of Medical and Dental Science, Tokyo Medical and Dental University (TMDU), Tokyo, Japan

**Keywords:** X-linked inhibitor of apoptosis, XIAP deficiency, SLAM-associating protein, SAP deficiency, Epstein-Barr virus, Hemophagocytic lymphohistiocytosis

## Abstract

X-linked lymphoproliferative syndrome (XLP) is a rare primary immunodeficiency disease that can be divided into two types: SAP deficiency (XLP1) and XIAP deficiency (XLP2), caused by mutations in the *SH2D1A* and *XIAP* genes, respectively. Few cases of XLP (particularly XIAP deficiency) have been reported in mainland China; hence, little is known about the characteristics of Chinese patients with XLP. We identified 13 and 7 patients with SAP and XIAP deficiency, respectively, in our center. Of our 20 patients, 19/20 (95%) presented with disease symptoms at a very early age: six in infancy and 13 in childhood. One XIAP- and three SAP-deficient patients died, while 3/7(42.9%) and 4/13(30.8%), respectively, developed hemophagocytic lymphohistiocytosis (HLH). Epstein-Barr virus (EBV) infection was significantly more common in SAP-deficient 10/13 (76.9%) than XIAP-deficient 2/7 (28.6%) patients, as was hypogammaglobulinemia (10/13 (76.9%) vs. 1/7 (14.3%)). None of the seven XIAP-deficient patients had colitis or lymphoma. Nine SAP-deficient patients and five XIAP-deficient patients showed markedly deficient SAP and XIAP expression, respectively, in lymphocytes. Significantly reduced levels of switched memory B cells were observed in six SAP-deficient patients with persistent hypogammaglobulinemia. One of 13 (7.7%) SAP-deficient patients and 1 of 7 (12.3%) XIAP-deficient patients have received HSCT treatment and are now alive and well; the other alive patients were waiting for HSCT. We also summarized clinical, genetic, and immunological characteristics of all 55 patients (including our 20 patients) reported in the literature in mainland China today.

*Conclusion*: The overall characteristics of SAP deficiency in mainland China were consistent with those in previous reports, whereas manifestations of XIAP deficiency varied significantly. None of inflammatory bowel disease (IBD) has been reported among XIAP-deficient patients in our center; however, whether Chinese XIAP-deficient patients will develop colitis in the future warrants further investigation. HSCT is the only curative therapy for XLP and this therapy should be urgently considered.

**Table:** 

**What is Known:** • *SAP and XIAP deficiencies share common clinical feature*, *HLH*, *whereas they also have their own specific manifestations*. • *IBD affects 25*–*30% of XIAP-deficient patients*, *which has been reported in other countries especially in European country and Japan*.	
**What is New:** • *This is the largest patient cohort study of XLP in China.* • *We firstly summarized the clinical features and outcomes of Chinese XIAP-deficient patients and found only 1 in 22 patients developed IBD and diet background may contribute to it; Asian SAP-deficient patients carrying SH2D1A R55X mutation were more prone to HLH*.

**Electronic supplementary material:**

The online version of this article (10.1007/s00431-019-03512-7) contains supplementary material, which is available to authorized users.

## Introduction

X-linked lymphoproliferative syndrome (XLP) is a rare primary immunodeficiency disease characterized by extreme vulnerability to Epstein-Barr virus (EBV) infection, and with clinical features including hemophagocytic lymphohistiocytosis (HLH), lymphoproliferative disorder, and dysgammaglobulinemia. XLP was initially described in the 1970s [30], while the first causative pathogenic gene, *SH2D1A*, which encodes signaling lymphocyte-activation molecule–associating protein (SLAM-associating protein, SAP) was discovered in 1998 [8]. In 2006, a second causative gene, *XIAP*, was found in some XLP-like patients without *SH2D1A* gene mutations [31]. Hence, XLP can be divided into two types: SAP deficiency (XLP1), caused by mutations in *SH2D1A*, and XIAP deficiency (XLP2), due to mutations in *XIAP*. Although SAP and XIAP deficiencies share some common clinical features, such as HLH and dysgammaglobulinemia, they also have their own specific manifestations. XLP patients have high mortality rates, and hematopoietic stem cell transplantation (HSCT) is the only curative therapy.

XLP is estimated to affect approximately one per million males, although it may be underdiagnosed [36]. The clinical characteristics of patients with XLP worldwide have been widely reported; however, to date, few cases of XLP, particularly XIAP deficiency, have been described in mainland China [17]. Hence, information about Chinese patients with XLP is scarce and improved characterization of the clinical symptoms of SAP and XIAP deficiency in Chinese individuals could facilitate better understanding of these diseases. Here, we describe 13 SAP-deficient and 7 XIAP-deficient patients treated in our center. We will also summarize clinical, genetic, and immunological characteristics of all further patients reported in the literature in mainland China today.

## Material and methods

### Patients

Male patients presenting with presumed XLP phenotypes from 2010 to the end of 2018 were screened using capture next-generation sequencing (CNGS) and Sanger sequencing. Thirteen SAP-deficient patients from ten families and seven XIAP-deficient patients from six families were identified. Several of these patients have been previously reported [2, 21]. Clinical and laboratory data were collected from the patients’ medical records, including clinical manifestations, laboratory tests, treatments, and outcomes. The study protocol was approved by the Institutional Review Board of Chongqing Medical University. Written informed consent was obtained from all individual participants. After consent was obtained, 5–10 mL of venous blood was collected into heparin-containing syringes and transferred at room temperature to our laboratory for analysis within 24 h of collection. PubMed and Chinese database for articles published from January 2000 to December 2018 were searched by using the key words of “X-linked lymphoproliferative syndrome,” “SH2D1A,” and “XIAP.” The relevant literatures were reviewed. Unfortunately, we cannot contact the authors for further information.

### Gene and protein expression analyses

The *SH2D1A* and *XIAP* genes were screened for mutations by CNGS at Mygenostics (Beijing, China), as described previously [15]. All suspected mutations identified by CNGS were confirmed by Sanger sequencing. Briefly, DNA was isolated from peripheral blood samples using a DNA Mini Kit (Cat. 51306, Qiagen Inc.) and all exons and flanking regions of *SH2D1A* and *XIAP* were amplified by polymerase chain reaction (PCR). PCR products were sequenced directly using the BigDye Terminator mix (Applied Biosystems) and oligonucleotide primers. Sanger sequencing was performed on an ABI Prism 3100 fluorescent sequencer (Applied Biosystems). Protein expression was evaluated by flow cytometry and Western blotting, as described previously [2, 42].

### Analysis of lymphocyte subsets

Conventional lymphocyte subsets were analyzed as previously described [11]. Total B cells (CD19^+^) and the following B cell subsets were examined: switched memory B (CD19^+^CD27^+^IgD^+^), naïve B (CD19^+^CD27^−^IgD^+^), transitional B (CD19^+^CD24^+^CD38^+^), and plasmablast (CD19^+^CD24^−^CD38^+^) cells.

### Statistical analysis

Statistical analysis was performed using GraphPad Prism 7.0 software. The significance of differences was evaluated using the unpaired *t* test, nonparametric Mann-Whitney test, or Fisher’s exact test. *P* < 0.05 was considered significant.

## Results

### Clinical characteristics of patients with XLP

Thirteen SAP-deficient patients from ten families and seven XIAP-deficient patients from six families were included in this study. Clinical data are summarized in Tables 1 and 2. The majority of patients presented with disease symptoms at very early ages; six patients presented in infancy and 13 in childhood. Eight SAP-deficient patients and two XIAP-deficient patients had family histories of XLP. To date, three of the patients with SAP deficiency and one with XIAP deficiency have died: P4 died of intracranial hemorrhage at the age of 1 year, P7 died of gastrointestinal hemorrhage at the age of 3 years, P8 died due to lymphoma recurrence and brain metastasis at the age of 7 years, and P36.1 died of pneumorrhagia at the age of 4 years. P1 and P37 have received HSCT and are currently alive and well.

**Table 1:** Clinical features of patients with SAP deficiency in mainland China

Patient	Age at onset (years)	Age at diagnosis (years)	Family history	Clinical presentation	EBV	IVIG	Allogenic HSCT (years)	Outcome	Age at death or current age (years)	*SH2D1A* mutation (protein)	SAP expression	Ref.
P1	1.42	1.5	−	HLH, Hypo-γ	+	+	2.65	Alive	2.92	R55X	Deficient	This study
P2	1.08	5.5	−	Hypo-γ, LPD	+	+	−	Alive	8.5	R55X	Deficient	This study
P3.1	7.0	12.0	+	Hypo-γ, LPD	+	+	−	Alive	14.42	R55L	NE	This study
P3.2	12.0	19.0	+	Hypo-γ, LPD	Unknown	+	−	Alive	20.42	R55L	NE	This study
P4	1.25	1.33	−	HLH, Hypo-γ, encephalitis	+	+	−	Died of intracranial hemorrhage	1.43	L25fsX1	Deficient	This study
P5*	4.00	4.5	−	Recurrent IM	−	−	−	Alive	9.92	Y47fsX12	Deficient	This study
P6	2.08	3.00	+	HLH	−	−	−	Alive	8.67	V40fsX34	NE	This study
P7	3.25	3.28	−	HLH, encephalitis	+	−	−	Died of gastrointestinal hemorrhage	3.38	W64X	NE	This study
P8	5	6	+	Lymphoma, Hypo-γ	+	+	−	Died of lymphoma	7	R55X	Deficient	This study
P9.1	1	9.83	+	Hypo-γ, LPD	+	+	−	Alive	12.5	R55X	Deficient	This study
P9.2	1	16.67	+	Hypo-γ, LPD	+	+	−	Alive	19.33	R55X	Deficient	This study
P10.1	0.08	14.83	+	Hypo-γ, lymphoma	+	+	−	Alive	17.33	G93D	Deficient	This study
P10.2	0.5	4.75	+	Hypo-γ, LPD	+	+	−	Alive	7.08	G93D	Deficient	This study
P11	4.00	4.08	−	HLH, Hypo-γ	+	+	4.08	Alive	Unknown	L98P	NE	[37]
P12	0.74	0.82	−	HLH, encephalitis, MSOF	+	+	−	Died of MSOF	0.81	Y47fsX12	NE	[37]
P13	1.40	1.48	−	HLH, encephalitis, MSOF	+	+	−	Died of MSOF	1.5	Y54C	NE	[37]
P14	3.92	4.00	+	HLH, Hypo-γ, MSOF	NE	+	−	Died of MSOF	Shortly after diagnosis	indels,138_201+18delinsGGTGAAAGAGGGTG	NE	[17]
P15	3.00	5.00	+	Hypo-γ, lymphoma	+	+	−	Died of hepatic coma	Shortly after diagnosis	Y54X	NE	[17]
P16	0.92	9.00	−	Hypo-γ, LPD	NE	+	−	Alive	Unknown	Y7C	NE	[17]
P17	1.75	1.75	+	HLH	+	/	−	Unknown	Unknown	R55X	NE	[46]
P18	2.83	2.92	−	HLH, LPD	+	+	−	Alive	Unknown	S35TfsX44	NE	[13]
P19	0.71	0.75	+	HLH, LPD	+	+	−	Dead	0.75	T61TfsX19	NE	[13]
P20	1.21	1.25	+	HLH, LPD	+	+	−	Dead	1.25	E67D	NE	[13]
P21	1.13	1.17	−	HLH, LPD	+	+	−	Dead	1.71	c.199_201+23del	NE	[13]
P22	1.5	1.5	/	HLH	NE	/	−	Unknown	Unknown	Y100X	NE	[22]
P23	3.0	3.0	/	HLH	NE	/	−	Unknown	Unknown	L31Rfs50X	NE	[45]
P24	/	0.67	/	HLH	/	/	/	Unknown	Unknown	R55X	NE	[6]
P25	/	1.0	/	HLH	/	/	/	Unknown	Unknown	c.199_201+23del	NE	[6]
P26	/	1.0	/	HLH	/	/	/	Unknown	Unknown	Y47VfsX21	NE	[6]
P27	/	1.0	/	HLH	/	/	/	Unknown	Unknown	Del. Exon1-4	NE	[6]
P28	/	4.0	/	HLH	/	/	/	Unknown	Unknown	Y76X	NE	[6]
P29	/	5.0	/	HLH	/	/	/	Unknown	Unknown	Del. Exon1-4	NE	[6]
P30	/	3.25	/	HLH	/	/	/	Unknown	Unknown	W64X	NE	[5]
P31	/	0.97	/	HLH	/	/	/	Unknown	Unknown	Y54X	NE	[5]
P32	/	0.95	/	HLH	/	/	/	Unknown	Unknown	R55X	NE	[5]

*EBV*, Epstein-Barr virus; *IVIG*, intravenous immunoglobulin; *HSCT*, hematopoietic stem cell transplantation; *Hypo-γ*, hypogammaglobulinemia; *LPD*, lymphoproliferative disease; *IM*, infectious mononucleosis; *HLH*, hemophagocytic lymphohistiocytosis; *MSOF*, multisystem organ failure; *NE*, not examined; +, positive; −, negative; /, no information; *fs*, frameshift; *del*, deletion; *ins*, insertion; *ref*, reference

**Table 2:** Clinical features of patients with XIAP deficiency in mainland China

Patient	Age at onset (years)	Age at diagnosis (years)	Family history	Clinical presentation	Recurrent HLH	Splenomegaly	Hypo-γ	Colitis	EBV	Allogenic HSCT (years)	Current age (years)	*XIAP* mutation (protein)	XIAP expression	Ref
P33	5.33	5.5	−	Recurrent respiratory infection	−	−	+	−	−	−	7.75	A321G	Normal	This study
P34#	0.67	1	−	HLH	+	+	NE	−	NE	−	3.08	Y75C	Deficient	This study
P35	2.75	3.75	−	Neutropenia	−	−	−	−	−	−	4.92	D367G	Deficient	This study
P36.1	0.04	1.92	+	Leukocytosis, thrombocytopenia	−	+	−	−	−	−	Died of pneumorrhagia	A321G	Deficient	This study
P36.2	−	12	+	Asymptomatic	−	−	−	−	NE	−	12.5	A321G	NE	This study
P37#	4.33	4.42	−	HLH	−	+	−	−	+	4.77	5.25	W317X	Deficient	This study
P38#	5	5.33	−	HLH	+	+	−	−	+	−	12.42	Del. Exon 4	Deficient	This study
P39	0.08	1.33	−	HLH	+	+	+	−	−	−	Unknown	T32fsX	NE	[17]
P40	0.08	0.25	−	HLH, MOSF	−	+	+	−	−	−	Died of MOSF	D367G	NE	[37]
P41	4	4.58	−	HLH	/	/	/	−	+	−	Unknown	c.1099 + 2T>C	NE	[44]
P42	5.8	5.8	−	HLH	−	+	+	−	−	6.05	Alive and well after HSCT	N341fsX348	NE	[16]
P43	5.1	6.1	−	HLH, Crohn’s disease	+	+	−	+	−	−	Unknown	G304X	NE	[41]
P44	/	0.33		HLH	/	/	/	/	/	/	/	T308IfsX23	NE	[6]
P45	/	1		HLH	/	/	/	/	/	/	/	E349del	NE	[6]
P46	/	1		HLH	/	/	/	/	/	/	/	Del.Exon3	NE	[6]
P47	/	2		HLH	/	/	/	/	/	/	/	D368EfsX23	NE	[6]
P48	/	2		HLH	/	/	/	/	/	/	/	R443H	NE	[6]
P49	/	2		HLH	/	/	/	/	/	/	/	R443H	NE	[6]
P50	/	4		HLH	/	/	/	/	/	/	/	Exonic deletion	NE	[6]
P51	/	5		HLH	/	/	/	/	/	/	/	c.1099+2T>C	NE	[6]
P52	/	6		HLH	/	/	/	/	/	/	/	D130GfsX11	NE	[6]
P53	/	28		HLH	/	/	/	/	/	/	/	R443H	NE	[6]

*EBV*, Epstein-Barr virus; *HSCT*, hematopoietic stem cell transplantation; *Hypo*-*γ*, hypogammaglobulinemia; *HLH*, hemophagocytic lymphohistiocytosis; *MSOF*, multisystem organ failure; *NE*, not examined; *fs*, frameshift; *del*, deletion; *ins*, insertion; #, novel mutation; *ref*, reference; +, positive; −, negative; /, no information

The rate of HLH occurrence in patients with SAP deficiency was 4 in 13 (30.8%), while that in XIAP deficiency was three in seven (42.9%). Concerning the prognosis of patients with XLP with HLH, fatal outcomes, such as neurologic involvement, were more frequently observed in patients with SAP deficiency than in those with XIAP deficiency; 2 of 13 (15.4%) SAP-deficient patients developed HLH with CNS symptoms and died soon after diagnosis, while in XIAP-deficient patients, all three patients with HLH lacked CNS symptoms and remain alive. In addition, HLH relapses appear to be more common in patients with XIAP deficiency, since all patients with HLH and XIAP deficiency experienced relapse. EBV infection was significantly more common in patients with SAP deficiency (10 of 13, 76.9%) than in XIAP-deficient patients (two of seven, 28.6%). Notably, all XIAP-deficient patients who presented with HLH had encountered EBV. Hypogammaglobulinemia was significantly more frequent in SAP-deficient patients (10 of 13, 76.9%) than in those with XIAP deficiency (one of seven, 14.3%). All patients with hypogammaglobulinemia were administered intravenous immunoglobulin (IVIG) substitution treatment; however, due to financial difficulties, only one SAP-deficient patient received IVIG regularly. Transient hypogammaglobulinemia was observed in one of seven (14.3%) XIAP-deficient patients, whereas 7 of 13 (53.8%) SAP-deficient patients presented with persistent hypogammaglobulinemia. Among SAP-deficient patients, 2 of 13 (15.4%) developed EBV-positive Burkitt lymphoma, while no XIAP-deficient patient presented with lymphoma. Further, four of seven (57.1%) patients with XIAP deficiency exhibited recurrent fever accompanied by splenomegaly as the first symptom of XLP. Among those four patients, three (75%) have developed HLH to date. None of our seven patients with XIAP deficiency, or their carrier mothers, developed colitis or other intestinal manifestations; however, one patient suffered from diarrhea for 3 days and recovered soon after symptomatic treatment.

### Analysis of gene and protein expression in patients with XLP

**Fig. 1:**
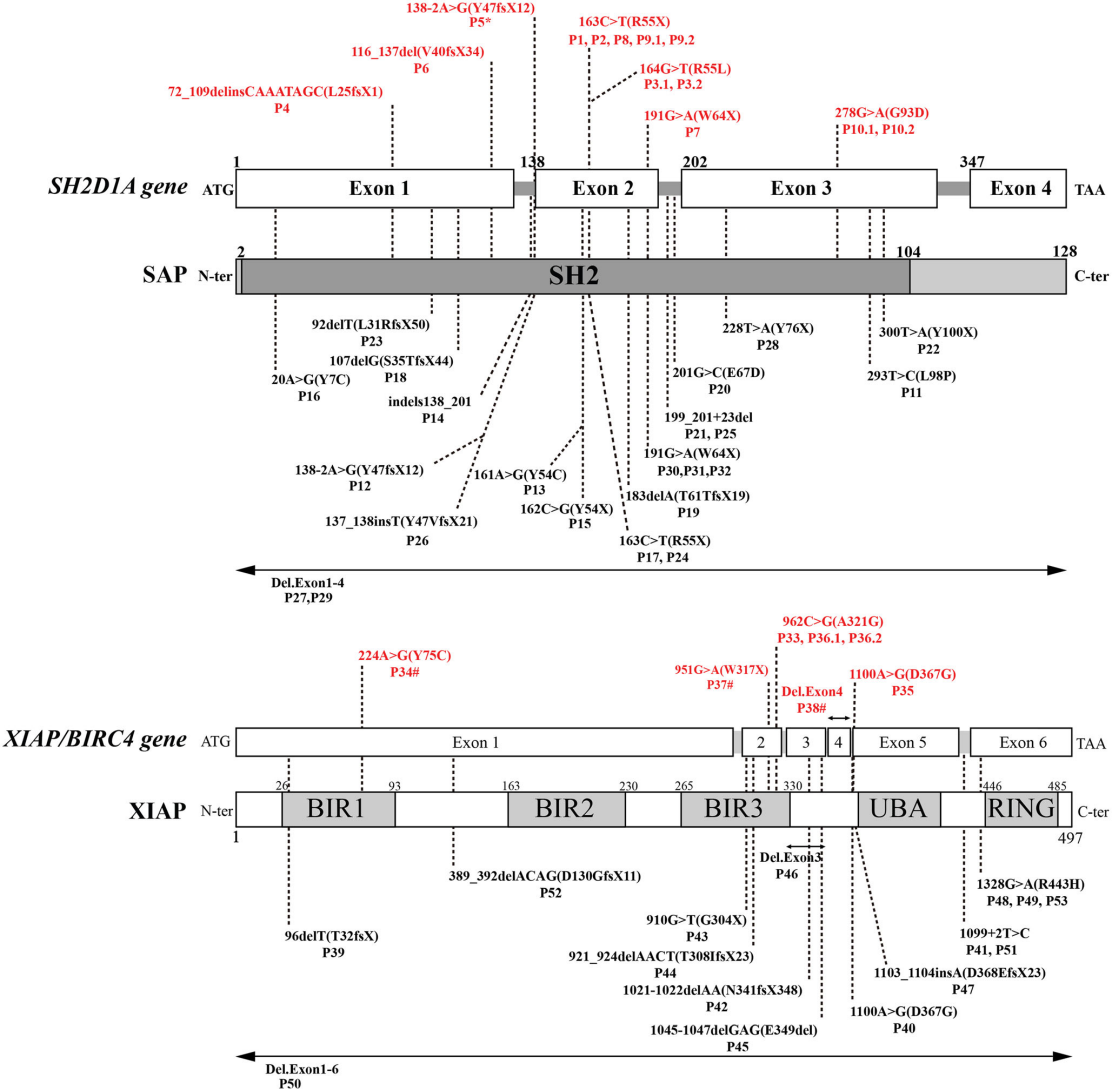
*SH2D1A* gene mutations in patients from China and their consequences for the SAP protein. Red text indicates patients diagnosed at our center, and black text represents patients diagnosed at other centers. Numbers above boxes representing exons indicate cDNA positions of exon boundaries. *XIAP*/*BIRC4* gene mutations in patients from China and their consequences for the XIAP protein. Red text indicates patients diagnosed at our center, and black text represents patients diagnosed at other centers. Numbers above boxes representing exons indicate cDNA positions of exon boundaries

We screened for *SH2D1A* and *XIAP* mutations in patients from all unrelated families (Fig. 1) and compared the data with those available in the US National Center for Biotechnology Information database (http://www.ncbi.nlm.nih.gov/SNP), to detect single-nucleotide polymorphisms. Four missense, six nonsense, and three splicing mutations were identified in patients with SAP deficiency. Seven mutations, five missense, one nonsense, and one deletion were identified in the *XIAP* gene; three of these (p.Y75C, p.W317X, del. Exon 4) were novel mutations. All mothers of patients were heterozygote carriers.

SAP and XIAP protein levels were analyzed in lymphocytes from patients and their family members by flow cytometry and/or Western blot analysis. All examined patients with SAP deficiency demonstrated markedly deficient SAP expression in lymphocytes. Five patients (P34, P35, P36.1, P37, and P38) had reduced XIAP levels, whereas XIAP levels were normal in the lymphocytes of one patient (P33).

### Immunological characteristics of patients with XLP

To evaluate the immunologic characteristics of patients with SAP deficiency and persistent hypogammaglobulinemia, we analyzed B lymphocyte subsets. As shown in supplementary Table, the total number of B cells in SAP-deficient patients with hypogammaglobulinemia did not differ from that in healthy controls, whereas significantly reduced numbers of switched memory B cells were detected. The number of plasmablast was also decreased in four of seven (57.1%) patients.

### Treatment of patients with XLP

HSCT is now the only curative treatment for patients with SAP or XIAP deficiency. In our center, 1 of 13 (7.7%) SAP-deficient patients and 1 of 7 (12.3%) XIAP-deficient patients have received HSCT treatment. Patient 1 received HSCT treatment in Children’s Hospital of Chongqing Medical University 14 months after he was diagnosed. He remained in full remission of HLH at the time of HSCT. A reduced intensity myeloablative conditioning (MAC) regimen was performed before HSCT and then the patient received fully matched unrelated peripheral blood stem cells. Now, patient 1 was alive and remained free of disease. Patient 37 suffered from severe HLH and received HSCT treatment 55 days after he was diagnosed in other hospital; thus, all we know is that the patient received half-matched peripheral blood stem cells from his father. He developed acute vascular rejection after HSCT, but after further treatment, he is now alive and well. The other alive patients were still waiting for HSCT with the majority on immunoglobulin replacement therapy because of the limited resources of donors matched to his/her HLA genotype.

## Discussion

To date, 130 different *SH2D1A* mutations (http://www.hgmd.cf.ac.uk/ac/gene.php?gene=SH2D1A) and 98 *XIAP* mutations (http://www.hgmd.cf.ac.uk/ac/gene.php?gene=XIAP) have been reported worldwide. In China, very few genetically characterized cases of XLP have been reported to date. The largest cohort of SAP-deficient patients recently reported in China comprised 19 patients [17], while reports of XIAP-deficient patients are very limited. Herein, we describe 13 patients with SAP deficiency and seven with XIAP deficiency treated in our center and summarize data from 33 SAP-deficient and 22 XIAP-deficient patients (including our 20 patients) reported in mainland China to date.

### Spectrum of mutation analysis and associated phenotype

The *SH2D1A* gene mutation, p.R55X, has been identified in 8/35 (22.9%) Chinese patients with SAP deficiency, indicating that this is a hotspot mutation in China. Among seven patients carrying p.R55X mutations, three manifested with HLH, one had lymphoma, and the remaining three patients primarily presented with hypogammaglobulinemia. Six patients with p.R55X mutation were reported in one Japanese cohort [19], four of whom manifested with HLH and died, while the other two had hypogammaglobulinemia; however, among cases carrying p.R55X mutations reported in Western countries, except for four cases with HLH as the main manifestation [3, 20, 25], the majority presented without this condition. Morra et al. [26] reported four members of a family with the p.R55X mutation affected by a variety of progressive immunoglobulin abnormalities [14]. Parolini et al. [29] reported three members in one family with p.R55X mutations, one of whom had histiocytic lymphoma, one non-Hodgkin lymphoma, and the other severe hepatitis alone. Talaat et al. [38] reported that two patients with p.R55X mutations developed lymphocytic vasculitis involving the central nervous system. These data suggest that patients in Asian countries carrying p.R55X mutations are prone to development of HLH, compared with patients in Western countries with this mutation.

Among 22 Chinese patients with XIAP deficiency, most had mutations located in the BIR3 and UBA domains of the XIAP protein, suggesting that these domains are *XIAP* mutation hotspots in patients from mainland China, consistent with the domains containing mutations hotspots previously reported in other countries [1, 35].

### Age of onset and diagnosis

In this study, the age of onset for both SAP-deficient and XIAP-deficient patients was very early; nevertheless, the time from onset to diagnosis varied greatly. The reduced diagnosis interval in XIAP deficiency is likely closely related to the recently acquired deeper understanding of these diseases by Chinese doctors. Moreover, the rapid development of gene sequencing technology in China in recent years has facilitated the identification of pathogenic genes.

### HLH

The major clinical manifestations of HLH in Chinese patients with XLP were similar to those previously reported [33]. In general, HLH occurred in both SAP-deficient and XIAP-deficient patients; however, XIAP-deficient patients developed HLH more frequently, relative to those with SAP deficiency. Nevertheless, HLH with neurologic involvement was more frequent in patients with SAP deficiency, with fatal outcomes. These data indicate that HLH is more likely to be severe and fatal in patients with SAP deficiency than in those with XIAP deficiency.

### EBV infection

EBV infection is reported to be a trigger for HLH in patients with XLP. Marsh [23] reported that 30% of ten XIAP-deficient patients with HLH were EBV-positive, while in a Japanese cohort, four of six (66.7%) XIAP-deficient patients presenting with HLH were associated with EBV, and the EBV infection rate was as high as 81.8% (27/33) in SAP-deficient patients [19]. Moreover, all the SAP-deficient patients with HLH were EBV-positive, whereas HLH developed in XIAP-deficient patients in the absence of EBV infection. These findings indicate that, even in the absence of EBV infection, HLH can develop in XIAP-deficient patients, and that EBV infections are more frequent in SAP-deficient than XIAP-deficient patients. The increased rate of EBV-associated HLH may be related to the high prevalence of EBV in the Asian population.

### IBD

Inflammatory bowel disease (IBD) is also a prevalent phenotype affecting 25–30% of XIAP-deficient patients, which has been reported in many other countries [35]. This phenotype is even more severe than HLH in some XIAP-deficient patients. XIAP is critical for signaling downstream of the Crohn’s disease susceptibility protein and nucleotide-binding oligomerization domain-containing 2 (NOD2) and essential for signal transduction via both NOD1 and NOD2, which are intracellular pattern recognition receptors, involved in innate immune host defenses [10]. XIAP deficiency is now considered an important cause of IBD; however, in the present study, only 1 of 22 (4.5%) Chinese XIAP-deficient patients developed IBD, while no other patients or carrier mothers suffered from colitis or other intestinal manifestations. Damgaard et al. [10] demonstrated that XIAP-BIR2 mutations abolish the XIAP-RIPK2 interaction, resulting in impaired ubiquitylation of RIPK2 and recruitment of linear ubiquitin chain assembly complex (LUBAC) to the NOD2 complex, which may explain why only one Chinese XIAP-deficient patient had IBD manifestations, since none of them had XIAP-BIR2 mutations. Nevertheless, Aguilar et al. [1] reported that early-onset IBD is a frequent clinical manifestation in patients with XIAP deficiency, not associated with mutations in a particular XIAP domain. In addition, available data indicate that environmental background (particularly diet background) may contribute to the manifestation of IBD in XIAP-deficient patients. In Japan, 29% XIAP-deficient patients are reported to develop IBD and the prevalence is rising due to increasing consumption of Westernized diets since Westernized diet–associated gut microbial dysbiosis is the most ubiquitous environmental factor in IBD [7, 18]. In China, since the food style in China becomes more Westernized, IBD associated with XIAP-deficient patients will be increasing observed. Meanwhile, some patients with XIAP deficiency who show IBD only may be missed.

### Lymphoma

Approximately 30% of SAP-deficient patients are reported to develop lymphoma; however, no XIAP-deficient patients with lymphoma have been reported to date [33]. Notably, before P38 was diagnosed with XIAP deficiency by gene sequencing, he was pathologically diagnosed with T cell lymphoma and received two rounds of CHOP chemotherapy; however, considering that there have been no reports of XIAP deficiency with lymphoma to date, and that the condition of P38 is generally improving, we excluded the diagnosis of lymphoma and considered that the patient had lymphoid hyperplasia. This suggests that the diagnosis of lymphoma in patients with XLP requires careful consideration by experienced pathologists and immunologists. In the present study, 3/24 (12.5%) SAP-deficient patients developed B cell non-Hodgkin lymphoma, and finally died of the condition. Nevertheless, 0 of the 12 XIAP-deficient patients developed lymphoma, consistent with previous reports. XIAP is ubiquitously expressed and its levels are significantly increased in cancer cells. Originally, the function ascribed to XIAP was anti-apoptotic activity; hence, loss of XIAP protein may protect patients from lymphoma [32].

### Hypogammaglobulinemia

Another important feature of SAP and XIAP deficiency is hypogammaglobulinemia, which is reported in up to 50% of SAP-deficient and 16% of XIAP-deficient patients [33]. As shown in Tables 3 and 4, in the present study, hypogammaglobulinemia was diagnosed in 14 of 24 (58.3%) SAP-deficient and 4 of 12 (33.3%) XIAP-deficient patients. Notably, SAP-deficient patients had persistent hypogammaglobulinemia, whereas the condition was transitory in patients with XIAP deficiency. SAP is critical for the generation and maintenance of long-term humoral immune responses [9], and the number of peripheral blood B cells was normal in SAP-deficient patients with hypogammaglobulinemia, whereas naïve B cell differentiation was impaired. Further investigation of one patient with XIAP deficiency who only exhibited neutropenia is warranted. The role of XIAP in human neutrophils remains unclear; Wicki [39] reported that loss of XIAP facilitates the switch to TNFα-induced necroptosis in mouse neutrophils; hence, the cause of neutropenia in our patient deserves further study.

**Table 3:** Comparison of clinical characteristics of patients with SAP deficiency in mainland China with those of patients from other countries

	Our center (13)		China (35)		Kanegane (33) [19]		Seemayer (272) [34]		Booth (91) [4]	
	Incidence	Mortality	Incidence	Mortality	Incidence	Mortality	Incidence	Mortality	Incidence	Mortality
HLH	4/13 (30.8%)	2/4 (50%)	25/33 (75.8%)	8/13 (61.5%)	18/33 (55%)	16/18 (89%)	157/272 (58%)	127/132 (96%)	32/91 (35.2%)	21/32 (65.6%)
Lymphoma or LPD	8/13 (61.5%)	1/8 (12.5%)	#14/24 (58.3%)	5/14 (35.7%)	7/33 (21%)	3/7 (43%)	82/272 (30%)	46/71 (65%)	22/91 (24.2%)	2/22 (9%)
Hypo-γ	10/13 (76.9%)	2/10 (20%)	#14/24 (58.3%)	4/14 (35.7%)	12/33 (36%)	4/11 (36%)	84/272 (31%)	34/75 (45%)	46/91 (50.5%)	6/46 (13.0%)

^#^Among 33 Chinese SAP-deficient patients, nine patients with HLH were reported with only gene mutation information and five patients were described in non-English papers

*LPD*, lymphoproliferative disease; *HLH*, hemophagocytic lymphohistiocytosis; *Hypo-γ*, hypogammaglobulinemia

**Table 4:** Comparison of clinical characteristics of patients with XIAP deficiency in mainland China with those of patients from other countries

	Our center (7)	China (22)	Japan [1, 27] (17)	France [31, 33] (35)	Germany [1, 12, 35, 43] (33)	USA [23, 40] (11)	England [31, 33] (9)
HLH	3/7 (42.9%)	18/22 (81.8%)	11 (64.7%)	25 (71.4%)	12 (36.4%)	7 (63.6%)	1 (11.1%)
Recurrent HLH	3/7 (42.9%)	# 4/12 (33.3%)	10 (58.8%)	/	/	/	/
EBV infection-associated HLH	2/7 (28.6%)	# 3/12 (25%)	4 (23.5%)	20 (57.1%)	8 (24.2%)	4 (36.4%)	1 (11.1%)
Splenomegaly	4/7 (57.1%)	# 8/12 (66.7%)	6 (35.3%)	18 (51.4%)	19 (57.6%)	4 (36.4%)	6 (66.7%)
Hypogammaglobulinemia	1/7 (14.3%)	# 4/12 (33.3%)	4 (18.2%)	4 (11.4%)	5 (15.2%)	2 (18.2%)	3 (33.3%)
Lymphoma	0/7 (0%)	# 0/12 (0%)	0 (0%)	0 (%)	0 (0%)	0 (0%)	0 (0%)
Colitis	0/7 (0%)	# 1/12 (8.3%)	6 (35.3%)	8 (22.8%)	10 (30.3%)	4 (36.4%)	3 (33.3%)

^#^Among 22 Chinese XIAP-deficient patients, ten patients with HLH were reported with only gene mutation information and one patient was described in non-English paper

/, no information; *HLH*, hemophagocytic lymphohistiocytosis; *EBV*, Epstein-Barr virus

### Treatment and prognosis

HSCT is the only curative treatment for patients with SAP or XIAP deficiency. Given the poor prognosis of many patients with SAP deficiency who have not undergone HSCT therapy, it is imperative that such patients are transplanted as soon as possible following genetic diagnosis, regardless of their clinical manifestations. Nevertheless, HSCT in XIAP deficiency is controversial. Initially, HSCT in XIAP deficiency was associated with poor prognosis; however, with the application of reduced intensity conditioning before HSCT, successfully treated cases are increasingly being reported [24, 28]. In the present study, 2 of 24 (8.3%) SAP-deficient patients and 2 of 12 (16.7%) XIAP-deficient patients received HSCT treatment and are now alive and well. Where possible, HSCT should be considered a necessary option to ensure survival and quality of life in patients with XLP syndrome.

### Limitation

Our study also has limitations. Firstly, our sample size is not very large. Secondly, owing to an ethical reason, we cannot obtain adequate blood samples for more functional experimental analysis.

## Conclusion

In general, we report the clinical, genetic, and immunological characteristics of 13 and 7 patients with SAP and XIAP deficiency, respectively, in our center, and review the literature related to XLP in China, so that more patients with XLP could be identified. Once XLP is confirmed, HSCT should be urgently considered.

## Electronic supplementary material

ESM 1 (DOCX 17 kb)

## Abbreviations

EBV: Epstein-Barr virus
Hypo-γ: Hypogammaglobulinemia
HLH: Hemophagocytic lymphohistiocytosis
HSCT: Hematopoietic stem cell transplantation
IVIG: Intravenous immunoglobulin
PID: Primary immunodeficiency
SLAM: Signaling lymphocyte-activation molecule
SAP: SLAM-associated protein
XLP: X-linked lymphoproliferative syndrome
XIAP: X-linked inhibitor of apoptosis
