# Supplementary material for: X-linked lymphoproliferative syndrome in mainland China: review of clinical, genetic, and immunological characteristic
Source: Eur J Pediatr. 2019 Nov 21;179(2):327–38. doi: 10.1007/s00431-019-03512-7 (PMC6970958; doi:10.1007/s00431-019-03512-7)
Supplement: Supplementary file 1 — (DOCX 17 kb) [file 431_2019_3512_MOESM1_ESM.docx]

**Supplementary Table Immunological features of SAP-deficient patients with hypogammaglobulinemia**

|  | B-lymphocyte subset | | | | | |  | | Ig level | | | | |
| --- | --- | --- | --- | --- | --- | --- | --- | --- | --- | --- | --- | --- | --- |
| Patient | CD19+ B  (cells/µl) | Naïve B (cells/µl) | Memory B  (cells/µl) | Plasmablasts (cells/µl) | Transitional B (cells/µl) |  | | IgG  (g/L) ↓ | | IgA  (g/L) ↓ | IgM  (g/L) ↓ | IgE  (IU/mL) |  |
| P1 | 1113.0  (461–1456) | 1042.9  (323–1089) | 4.2 ↓  (26–124) | 3.1 ↓  (4–63) | 219.3 ↑  (35–172) |  | | 3.05 | | 0.134 | 0.202 | 3.8 |  |
| P2 | 592.4  (280–623) | 563.3 ↑  (147–431) | 7.1 ↓  (31–94) | 1.2 ↓  (4–28) | 53.9  (10–66) |  | | < 0.333 | | < 0.0667 | 0.07 | < 5.0 |  |
| P3.1 | / | / | / | / | / |  | | 0.74 | | < 0.0667 | 0.09 | 0.2 |  |
| P9.1 | 617.6 ↑  (216–536) | 568.8 ↑  (123–362) | 5.6 ↓  (28–89) | 0.6 ↓  (3–21) | 24.7  (7–37) |  | | < 0.333 | | < 0.0567 | < 0.0417 | 0.5 |  |
| P9.2 | 242.3  (203–476) | 224.6  (116–347) | 5.8 ↓  (20–86) | 0.7 ↓  (1–23) | 1.2 ↓  (4–37) |  | | < 0.333 | | < 0.0667 | 0.0950 | 0 |  |
| P10.1 | 414.2  (203–476) | 367.4  (203–476) | 7.0 ↓  (20–86) | 6.2  (1–23) | 6.2  (4–37) |  | | < 0.333 | | < 0.0667 | 0.1410 | 0.2 |  |
| P10.2 | 915.5 ↑  (280–623) | 866.0 ↑  (147–431) | 22.9 ↓  (31–94) | 10.1  (4–28) | 96.1 ↑  (10–66) |  | | 3.23 | | 0.195 | 0.296 | 0.8 |  |

**↑, increased; ↓, decreased**
